# Synthetic PET via Domain Translation of 3-D MRI

**DOI:** 10.1109/trpms.2022.3223275

**Published:** 2022-11-18

**Authors:** Abhejit Rajagopal, Yutaka Natsuaki, Kristen Wangerin, Mahdjoub Hamdi, Hongyu An, John J. Sunderland, Richard Laforest, Paul E. Kinahan, Peder E. Z. Larson, Thomas A. Hope

**Affiliations:** Department of Radiology and Biomedical Imaging, University of California at San Francisco, San Francisco, CA 94158 USA; Department of Radiation Oncology, University of New Mexico, Albuquerque, NM 87131 USA.; G.E. Healthcare, Waukesha, WI 53188 USA.; Department of Radiology, Washington University in St. Louis, St. Louis, MO 63130 USA.; Department of Radiology, Washington University in St. Louis, St. Louis, MO 63130 USA.; Department of Radiology, The University of Iowa, Iowa City, IA 52242 USA.; Department of Radiology, Washington University in St. Louis, St. Louis, MO 63130 USA.; Department of Radiology, University of Washington, Seattle, WA 98195 USA.; Department of Radiology and Biomedical Imaging, University of California at San Francisco, San Francisco, CA 94158 USA; Department of Radiology and Biomedical Imaging, University of California at San Francisco, San Francisco, CA 94158 USA

**Keywords:** Digital phantoms, full-convolutional neural networks, PET/MRI qualification, standardized uptake value (SUV) quantification

## Abstract

Historically, patient datasets have been used to develop and validate various reconstruction algorithms for PET/MRI and PET/CT. To enable such algorithm development, without the need for acquiring hundreds of patient exams, in this article we demonstrate a deep learning technique to generate synthetic but realistic whole-body PET sinograms from abundantly available whole-body MRI. Specifically, we use a dataset of 56 ^18^F-FDG-PET/MRI exams to train a 3-D residual UNet to predict physiologic PET uptake from whole-body T1-weighted MRI. In training, we implemented a balanced loss function to generate realistic uptake across a large dynamic range and computed losses along tomographic lines of response to mimic the PET acquisition. The predicted PET images are forward projected to produce synthetic PET (sPET) time-of-flight (ToF) sinograms that can be used with vendor-provided PET reconstruction algorithms, including using CT-based attenuation correction (CTAC) and MR-based attenuation correction (MRAC). The resulting synthetic data recapitulates physiologic ^18^F-FDG uptake, e.g., high uptake localized to the brain and bladder, as well as uptake in liver, kidneys, heart, and muscle. To simulate abnormalities with high uptake, we also insert synthetic lesions. We demonstrate that this sPET data can be used interchangeably with real PET data for the PET quantification task of comparing CTAC and MRAC methods, achieving ≤ 7.6% error in mean-SUV compared to using real data. These results together show that the proposed sPET data pipeline can be reasonably used for development, evaluation, and validation of PET/MRI reconstruction methods.

## I. Introduction

There is currently an unrealized potential for PET/MRI systems in synergistic and quantitative reconstructions that account for and leverage simultaneous data acquisition of PET, which provides functional tissue information, and MRI, which provides excellent anatomic information, to correct for artifacts, motion, and improve localization [1]. An example of one of the challenges is quantitative PET reconstruction, which requires accurate attenuation maps that are not directly measured by MRI. This affects the quantification of PET from reconstructed imagery, since the photon attenuation map is embedded in the forward system model. As a result, the development of novel attenuation correction methods and other advanced PET/MRI reconstructions requires real or realistic data, which can be difficult and/or expensive to obtain. With increased PET/MRI adoption, it is necessary to establish standards for the quality of reconstruction, which can vary based on subtleties of PET data collection, including scanner geometry and detector nonidealities, but also the choice of reconstruction algorithm, attenuation correction method, and patient anatomy (e.g., scattering and hyper-attenuation). Simulating the whole range of patient variability in terms of anatomy and patient-specific radiotracer uptake is infeasible, e.g., using purely digital phantoms and Monte Carlo simulation [2], [3], necessitating the acquisition of real patient PET data. For PET/CT systems, qualification methods are established by the American College of radiology (ACR) using qualitative evaluation of whole-body clinical scans and quantitative evaluation using a ACR PET Phantom, a cylinder based on the Jaszczak Deluxe Flangeless ECT phantom with the spheres removed, a PET faceplate composed of several fillable cylinders, and acrylic rods of various diameters [4]. PET reconstruction performance can also be measured using the NEMA phantom, which is composed of multiple fillable spheres and cylindrical inserts that aim to mimic attenuation and activity found in different parts of the human body [5].

Unfortunately, phantoms used for PET/CT are insufficient for evaluating PET/MRI reconstruction performance because they cannot evaluate modern MR-based attenuation correction (MRAC) methods that rely on detecting typical human anatomy from MRI data. These methods include vision-based atlas techniques [6], [7], [8], joint-reconstruction of attenuation and activity [9], [10], and direct prediction of pseudo-CT via deep learning-based domain translation [11], [12], [13], [14]. A physical PET/MRI phantom to evaluate reconstruction performance would require an anthropomorphic distribution of materials with properties that match both 511-keV photon attenuation as well as MRI properties of proton density, T1, and T2, which is extremely challenging especially for bone due to its high attenuation but rapid T2 decay rate [15].

Consequently, the standard approach to evaluating PET/MRI performance is to utilize human subject datasets acquired on PET, CT, and MR [11], [16]. This allows for a *relative* performance measure, by comparing the standardized uptake value (SUV) of MRAC-based PET reconstructions relative to PET reconstructions utilizing CT-based attenuation correction (CTAC) [16]. However, for sites to conduct such evaluations, numerous PET/CT/MR scans are required to characterize scanner and algorithm performance at operating points exhibiting natural imperfections that impact the physics of PET collection, such as those arising from detector characteristics, scattering, or unexpected attenuation [17]. This patient-specific data is expensive to collect, hindering new PET/MRI algorithm development that normally requires recollecting PET data.

**Fig. 1.**
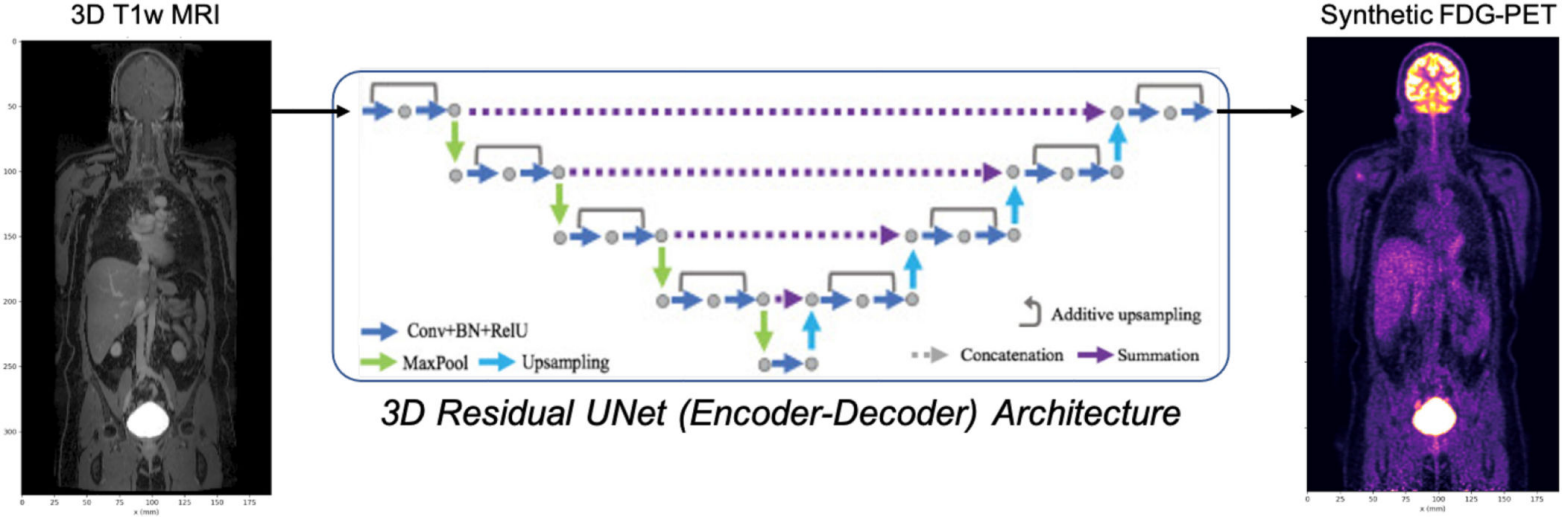
3-D residual UNet architecture for generating sPET from MRI, requiring only paired (registered) PET/MRI data without annotation.

In this article, we present a method for generating *synthetic* PET data using routinely collected and abundantly available MRI that naturally captures important scanner and detector imperfections, adapts to varied tracer distributions and anatomy, and allows for insertion of synthetic lesions. We believe this will allow for the creation of large and diverse synthetic data for development, evaluation, and validation of PET reconstruction algorithms. Our approach leverages recent work in deep learning-based domain translation using fully convolutional networks (FCNs) and in Section II we describe how to train a 3-D residual UNet to predict SUV-normalized synthetic PET (sPET) imagery from whole-body postconstrast T1-weighted MRI (Fig. 1). This requires only paired input and output image examples, and—crucially—no additional annotation or scanner geometry details. For this problem, we assume a supervised setting, where the absolute and relative error between the measured (reconstructed) and FCN-generated volumes provide a quantitative measure of performance, albeit at different scales that must be balanced. Note that an approach based purely on generative adversarial networks (GANs) is not desirable here, since we require the sPET volumes to correspond anatomically to the MRI volumes to support PET/MRI reconstruction research. To this end, in Section III, we show that the predicted sPET imagery can be forward projected to generate sPET time-of-flight (TOF) sinogram data that can be used interchangeably with real PET sinogram data in vendor-provided reconstruction algorithms. We further evaluate this capability for qualification research by performing a classical PET-SUV quantification experiment, comparing reconstructions with CT- and 2-point MR-Dixon-based AC maps, using both synthetic FDG-PET and measured FDG-PET sinograms. Our results show that evaluation using sPET can achieve < 8% quantification error in mean-SUV in synthetically inserted lesions compared real PET data (averaged over a several synthetic lesions in a cohort of patients), suggesting the wide applicability of domain-translated sPET for PET reconstruction algorithm development and qualification research. The role of synthetic lesions as proposed and demonstrated in this study is to provide methods for evaluation and optimization of image reconstruction algorithms. These algorithms continue to change and, with the introduction of deep learning/ AI methods for image reconstruction and denoising algorithms, many new parameters are being introduced and more robust methods for evaluation and optimization are needed to demonstrate the clinical impact of the image processing algorithms

### A. Prior Work

Prior work in deep learning-based domain translation has demonstrated that FCNs based on UNet-like encode-decoder architectures are widely applicable to a range of 2-D and 3-D cross-modality medical image translation tasks, including MRI-to-CT [11], PET-to-CT [18], and MRI-to-MRI [19]. These architectures are typically trained independently for each anatomical region (e.g., head, chest, and pelvis) of interest. For PET/MRI specifically, a major focus has been in MRI-to-CT domain translation for enhanced attenuation correction maps, which are ultimately combined with measured PET sinogram data for enhanced image reconstruction [11], [13], [18]. Recently, such architectures have been applied to the reconstruction and denoising of low-dose PET imagery, including using supervised [20], [21] and unsupervised [22] methods, and extensions to dynamic PET reconstruction [23]. In some cases, these image-enhancement techniques have been shown to successfully improve diagnostic interpretability [24].

In contrast to these works, the focus of this article is domain translation of whole-body MRI-to-PET *without* any initial PET data, i.e., to produce a novel image series we refer to as synthetic PET (sPET). While previously in-silico PET image generation has been explored using physics-based simulation tools such as GATE [25] with Monte Carlo techniques, such as PENELOPE [26] and SimPET [3] to reproduce realistic image quality, a predominant issue here is knowing realistic spatial distribution of physiologic PET uptake to seed the simulation. Our work addresses this issue by using a neural network to learn from real PET scans, such that realistic physiologic uptake can be inferred from abundantly available MRI. This is an important point since we do not believe sPET can accurately predict patient-specific functional information for diagnosis.

### B. Contributions

Thus, our contributions are as follows.

1. We introduce a deep learning method for generating whole-body 3-D sPET volumes from one or more routinely collected MRI series, including a balanced loss function that improves reconstruction of both low- and high-SUV regions.
2. We evaluate the utility of sPET in a downstream development task involving the quantification of PET SUV in images reconstructed using MR- and CTAC, demonstrating that sPET sinograms can be used seamlessly in place of real PET data for PET/MRI qualification with minimal impact to the observed quantification error in synthetically inserted lesions.

## II. Synthetic PET via Domain Translation

Although the physics and acquisition are fundamentally different, MRI and PET imagery share a great deal of structural similarity due to contouring of patient anatomy by physiologic uptake. This similarity can be exploited by FCNs to efficiently and *implicitly* implement the codebook $𝓒:{\mathbb{R}}_{\text{MR}}^{n}\to {\mathbb{R}}_{\text{PET}}^{n}$ mapping MRI to PET-SUV imagery using a cascade of nonlinear filters, avoiding explicit storage of input–output pairs (x, y) in a database. Note that this map 𝓒 describes a *statistical* relationship between MRI and PET, and not a causual or functional one. Besides being differentiable and amenable to backpropagation-based optimization using historical PET/MRI datasets, FCNs have strong spatial regularization properties that reduce degeneracy across image patches to create seamless and realistic anatomy-conforming 3-D PET imagery from MRI.

Here, degeneracy refers to the typical inconsistencies in the codebook arising from the fact that PET and MRI contain different (orthogonal) information about a patient. The inverse image ${𝓒}^{-1}(y)$ of a 3-D PET patch $y\in {\mathbb{Z}}_{\text{PET}}^{n}$ may not be unique, since different anatomical regions can experience the same uptake. Conversely, a given 3-D MR patch $x\in {\mathbb{R}}_{\text{MR}}^{n}$ may have multiple images $𝓒(x)\in {\mathbb{Z}}_{\text{PET}}^{n}$, corresponding to various patterns of PET uptake across individuals. Thus, the map 𝓒 is general, which frustrates conventional atlas and dictionary-based implementations that must keep track of this in ${\mathbb{R}}_{\text{MR}}^{n}$ [27]. In comparison, due to the supervised training process, FCNs naturally choose y←E[𝓒(x)] for sPET given input MRI x. In this respect, the task of predicting PET from MRI is distinct from approaches predicting full-dose PET imagery from low-dose PET imagery, since those models are responsible for enhancing the signal-to-noise ratio (SNR) of existing activity images [20], [21], rather than directly learning anatomy-conforming physiologic biodistributions of PET uptake.

### A. Assumptions

In this article, we assume the availability of historical PET/MRI datasets of patients receiving a calibrated (full) dose of the same PET radiotracer. Although the proposed method is applicable to varying dose levels, low-dose PET imagery exhibits lower SNR, and is therefore not ideal for training. In this work, we register scanner-reconstructed whole-body ^18^F-FDG-PET and post-contrast T1-weighted MRI volumes, collected on a 3.0 T ToF PET/MRI scanner (Signa, GE Healthcare, Waukesha WI), to the MRI image space and resample to 1-mm isotropic resolution using the ANTS toolbox interface provided via Nipype [28]. To increase the regularity and identifiability of MR structures, we apply contrast-limited adaptive histogram normalization to the resampled MRI volumes, using a kernel-size of 100 mm and clipping limit of 0.05 [29]. For consistency, we convert the raw PET intensity values (counts) to SUV using known radiotracer dose, half-life, positron fraction, elapsed time, and patient weight [30]. Finally, we split our dataset into 40 whole-body PET/MRI training exams, 16 whole-body PET/MRI testing exams, and 20 independent pelvic PET/MRI testing exams where corresponding CT was available (discussed in Section III). We make no explicit assumption of age, race, gender, or ailment, other than through the image characteristics of the acquired dataset.

### B. Model Architecture

By fiat, we choose a 3-D residual UNet architecture that combines the well-studied 2-D/3-D UNet [31] with residual (skip) connections [32] and convolutional upsampling (Fig. 1). In our implementation, we take a one-channel 3-D MRI volume as input, and employ 3 × 3 × 3 convolutional kernels followed by 2 × 2 × 2 maxpooling in each layer of the encoder (channel dimensions: [32, 64, 128, 256, 512]), and 3 × 3 × 3 convolutional upsampling kernels in each layer of the decoder (channel dimensions: [256, 128, 64, 32]), ultimately resulting in a one-channel 3-D output. This architecture can be adapted to multichannel inputs (multicontrast MRI) and outputs (multiple PET radiotracers and/or dose levels) by modifying the first and last layers of the network, respectively.

Inference is performed by breaking large whole-body MRI volumes into smaller overlapping volumetric patches with dimensions divisible by 32 (e.g., [128 × 128 × 128] mm, with 50% overlap) prior to applying the 3-D UNet, and taking the sample mean of the resulting outputs at each 3-D grid position to assemble the full whole-body volume. While the aforementioned resampling ensures MRI is processed at nearly native resolution to allow recognition of fine structural details, the PET groundtruth is considerably upsampled, especially in the z dimension. This can be remedied by resampling the predicted volumes to the native PET image space and resolution, e.g., prior to performing PET/MRI reconstruction (Section III).

### C. Learning

**Fig. 2.**
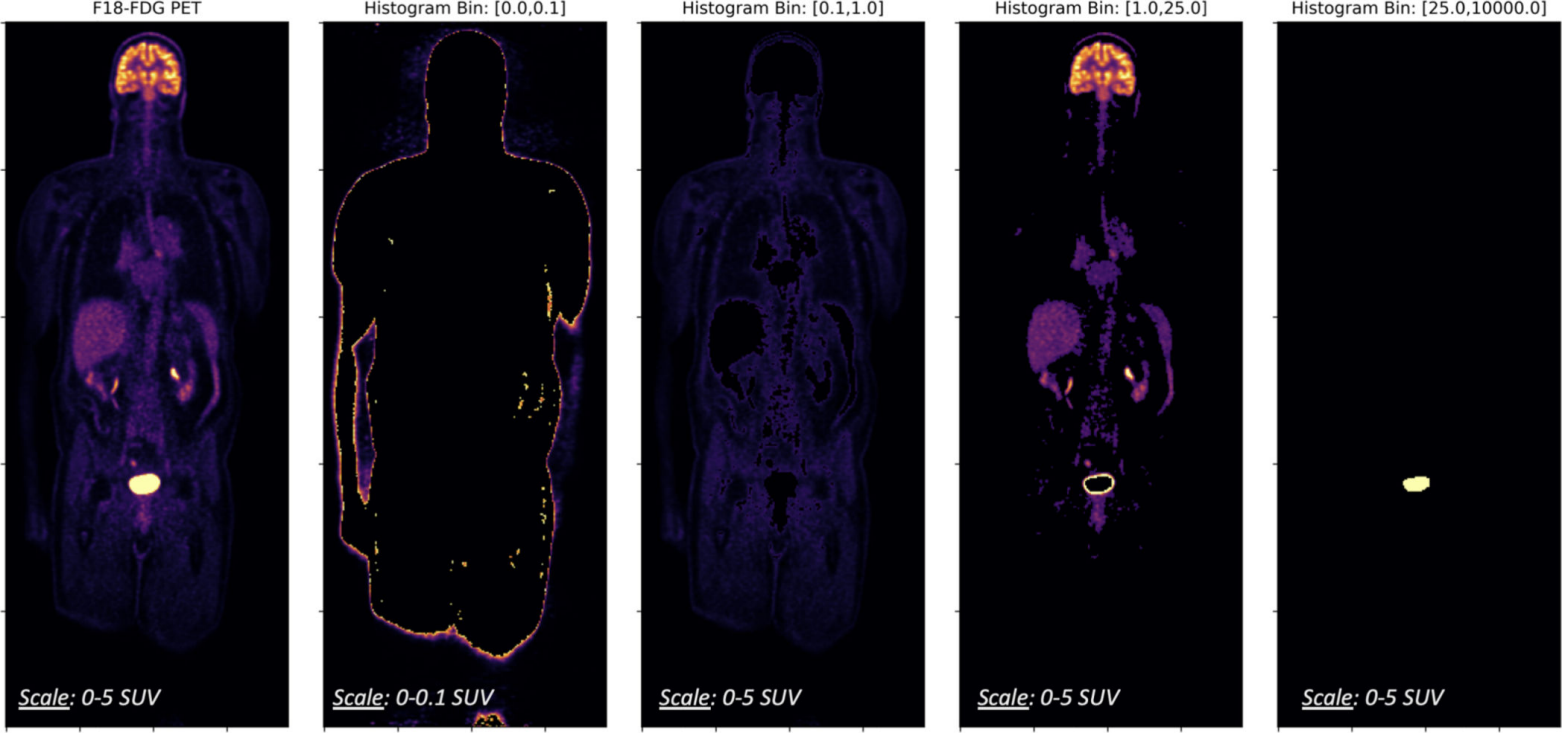
Histogram distribution of a whole-body PET exam reveals disparate levels of physiologic activity across different anatomy.

One of the primary challenges with domain translation of MRI to PET is maintaining high accuracy across the full dynamic range of PET. Although SUV scaling does provide a more consistent and intuitive numerical range, we find that explicit control in the objective function is required to prevent smoothing over suitable minima. For example, the histogram distribution of a whole body ^18^F-FDG-PET exam (Fig. 2) reveals that different tissues differ in the amount of physiologic uptake. For example, in the lungs, heart, and liver there is often increased activity between [1,4] SUV, and in regions, such as the bladder and brain the recorded SUV can be greater than 20. In particular, since we are interested in using the predictions of our model for PET quantification studies, we require high accuracy across all relevant scales. This precludes the use of simple p-norm objective functions, such as the mean absolute error (MAE), that may be dominated by the high absolute or relative error in one or more histogram bins.

To address this, we minimize the balanced objective

(1) $$J_{\text{total}}=J+\lambda J_{\text{LOR}}$$

where $J_{\text{LOR}}$ represents a regularization function with parameter λ, and J is a linear combination of absolute and relative errors across B different histogram bins, expressed as follows:

(2) $$J=\sum_{j=1}^{B}\frac{\alpha_{j}}{\left|h_{j}\right|}\sum 𝓔\left[h_{j}\right]+\sum_{k=1}^{B}\frac{\beta_{k}}{\left|h_{k}\right|}\sum \frac{𝓔\left[h_{k}\right]}{y\left[h_{k}\right]+\epsilon }$$

where 𝓔=|F(x)−y| is the conventional voxel-wise absolute error, x is the MRI input volume, y is the groundtruth PET volume, and F(x) represents the predicted synthetic sPET. In (2), $h_{j}$ represents an indicator variable selecting the voxels belonging to bin j of the B-bin histogram of y, and ϵ is chosen as 1e-3 to prevent overflow. The histogram bins (Fig. 2) and corresponding weights ($\bar{\alpha }=[1,1,1,1,0]$, $\bar{\beta }=[0,0,1,1,1]$) were chosen based on empirical observation to prevent domination of J by high absolute errors in high-SUV regions or by high relative errors in low-SUV regions. The intention of this flexible formulation with $\bar{\alpha }$ and $\bar{\beta }$ is to define a family of functionals that can be tailored to different patient datasets, PET tracers, and anatomic regions.

To further improve both the perceptual image quality and convergence, during training we integrate and compare the groundtruth PET y and the predicted sPET $\hat{y}=F(x)$ along random angles using a projection operator ${𝓡}_{\theta ,\phi }$, mimicking tomographic data collection in a uniform, isotropic attenuating media along hypothetical PET lines of response (LOR), as follows:

(3) $$J_{\text{LOR}}={\left\|{𝓡}_{\theta ,\phi }\cdot (F(x)-y)/(y+\epsilon )\right\|}_{2}.$$

In addition to tying together the performance of different tomographically related voxels, $J_{\text{LOR}}$ measures the error in the coarse *scale* of predictions on a line-by-line basis. For example, if a 3-D image patch shows little to no activity, ${\left\|{𝓡}_{\theta ,\phi }y\right\|}_{2}$ will be nearly zero, whereas a patch from a region with high uptake may yield either high- or low-valued ${\left\|{𝓡}_{\theta ,\phi }y\right\|}_{2}$. This improves convergence and combats overfitting by supervising the spatial distribution of sPET without explicit assumptions of patient anatomy.

For all results shown in this article, we used the Adam optimizer with an initial learning rate of 1e-4, weight decay of 1e-3, and effective batchsize of 16 [128×128×128] mm volumetric patches generated systematically (in a random order) from the aforementioned whole-body ^18^F-FDG-PET/MRI dataset.

To improve convergence during training, we defined a custom 3-D image patch sampler that performs round-robin sampling of different PET/MRI phenotypes present in the training dataset. These phenotypes were determined by first cataloging all the volumetric patches in the training dataset and computing their intensity histograms. Using k-means clustering (K=10), we computed a semantic grouping of these histograms that defined the different PET/MRI phenotypes that were sampled cyclically during model training.

### D. Image Quality Metrics

We measure the quality of predicted sPET using quantitative error metrics, including the MAE, mean relative absolute error (MRAE), and the 3-D structural similarity index measure (SSIM). For each exam we compute MAE over all voxels N, as follows:

(4) $$\text{MAE}=\frac{1}{N}\sum_{n}{\left\|y_{n}-{\hat{y}}_{n}\right\|}_{1}$$

while we compute MRAE only over voxels K of at least 0.1 SUV, as follows:

(5) $$\text{MRAE}=\frac{1}{K}\sum_{k}\frac{{\left\|y_{k}-{\hat{y}}_{k}\right\|}_{1}}{y_{k}}.$$

The 3-D-SSIM captures this information in a different way, accounting for differing scales and magnitudes through a measure of correlation within a 3-D window, as follows:

(6) $$\mathrm{SSIM}(x,y)=\frac{\left(2\mu_{x}\mu_{y}+c_{1}\right)\left(2\sigma_{xy}+c_{2}\right)}{\left(\mu_{x}^{2}+\mu_{y}^{2}+c_{1}\right)\left(\sigma_{x}^{2}+\sigma_{y}^{2}+c_{2}\right)}$$

where $\mu_{x}$ and $\sigma_{x}^{2}$ represent the mean and variance of volume x, $\mu_{y}$ and $\sigma_{y}^{2}$ represent the mean and variance of volume y, $\sigma_{xy}$ represents the covariance of x and y, and $c_{*}$ is chosen proportional to the dynamic range of pixel values [33].

### E. Results on Whole-Body ^18^F-FDG PET-MR Datasets

**Fig. 3.**
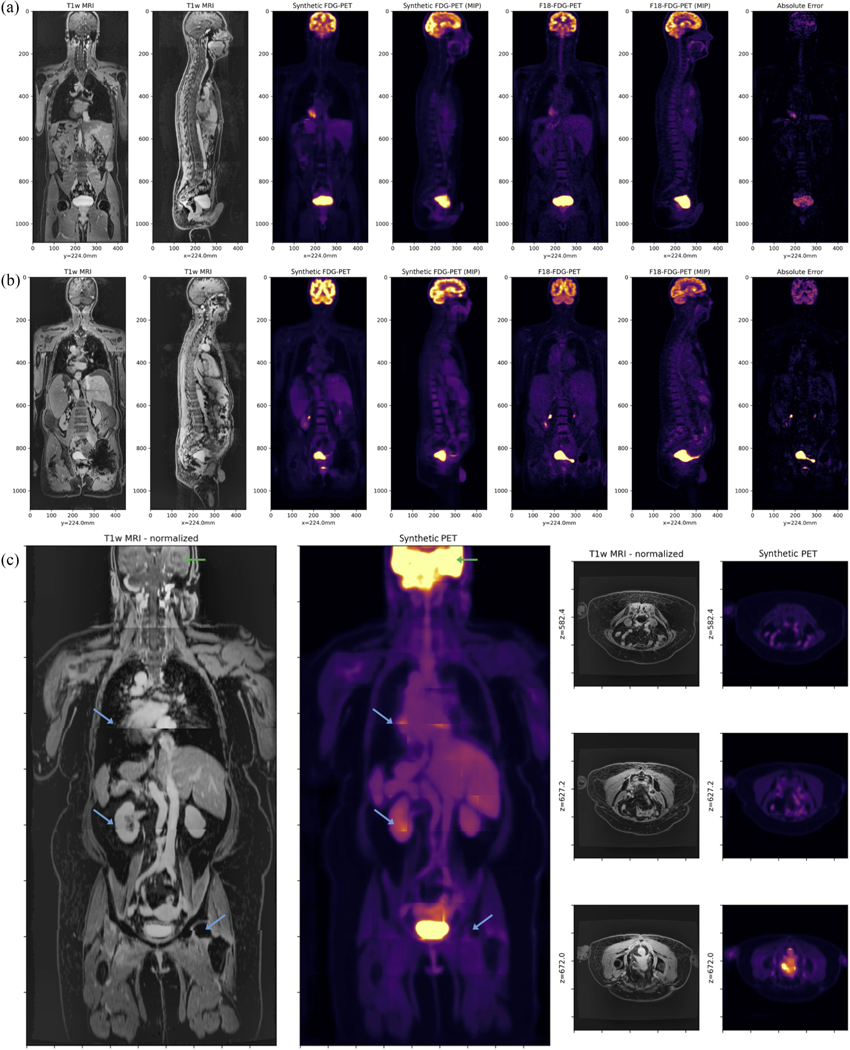
(a) and (b) Test-set evaluation of whole-body MR-based synthetic FDG-PET (sPET) in comparison to real ^18^F-FDG-PET/MRI. sPET mimics the typical physiologic uptake of FDG, showing high uptake in the brain and bladder as well as moderate uptake in liver, kidneys, heart, and muscle. High relative error with the real PET data is expected in many regions where there is typically high physiologic variability between subjects (e.g., tumors, heart, and bladder). While (a) and (b) represent patient exams from the intentionally withheld test set, (c) represents an exam from the additional validation set (with corresponding Pelvic PET/CT) exhibiting significant stitching artifacts (blue arrows) in the T1w-MRI between bed positions as well as loss in resolution in the head (green arrows). Various transverse slices in the abdomen are shown for comparison on the right of (c). Evaluation and inclusion of this exam in the validation cohort demonstrates that the proposed 3-D UNet is able to recover reasonable FDG-uptake even in the presence of significant domain shift, a common issue when applying deep learning algorithms to clinical data acquired on a different scanner, or with different imaging protocols and image quality checks.

We find that prediction of synthetic FDG-PET, domain translated from T1-weighted post-contrast MRI, works well despite the lack of salient tracer specific or functional information in MRI (Fig. 3). Numerical results comparing the effect of different training objectives on test-set performance is shown in Table I. Qualitative analysis reveals that physiologic uptake is predicted accurately and reconstructed seamlessly throughout the body without obvious spatial artifacts, except in regions where we expect variable uptake (e.g., heart and bladder). In the myocardium, for example, FDG-PET uptake depends on patient metabolism, which can vary across exams for even a single patient. Similarly, in the bladder PET uptake is often dependent on a patient’s water consumption and timing of voiding [34].

The MAE and MRAE results show that incorporation of both balanced histogram losses and tomographic projection-based losses can significantly reduce the quantitative error in the prediction of sPET from MRI. The SSIM results show that this reduction in error boosts the image quality of the sPET image relative to the real PET image. The inclusion of SSIM is important to assess the realness of sPET, in lieu of reporting MAE and MRAE within different organs and anatomical structures.

## III. PET Quantification Using Synthetic PET

PET/MRI quantification is important for establishing the accuracy and reproducibility of PET reconstructions when the photon attenuation maps are inferred entirely from MRI. As the error in PET/MRI reconstruction is composed of errors involving prediction of the attenuation map and errors involving the reconstruction (e.g., choice of the objective function), a standard approach is to measure the compound effect caused by the AC map by directly comparing PET volumes reconstructed with MRAC and CTAC voxel-wise and regionally [16], [35].

Specifically, we evaluate the applicability of our MR-derived sPET imagery for algorithm development by replicating an MRAC versus CTAC PET SUV quantification task using sPET data in place of real list-mode PET data. To achieve this we forward project sPET data into sinogram space using vendor-provided software that incorporates scanner geometry, detector response, and normalization.

### A. Reconstruction Model and Parameters

For time-of-flight PET (ToF-PET), the measured sinogram data is modeled within the forward model as follows [36]:

(7) $$y_{pt}=A_{pt}x+b_{pt}$$

where $y_{pt}$ represents the ToF projection data measured by the scanner, x is the PET image to be found, and the system matrix A models the probability of an event emitted in voxel m to be detected by detector pair p within the signed timing bin number t, summarizing the attenuation of the media along PET LoR, patient-scanner geometry, and detector efficiencies. $b_{pt}$ corresponds to the background counts of the timing bin t and detector pair p. For this model, a basic reconstruction approach is to solve the optimization problem

(8) $$\hat{x}=\underset{x}{\min}\|Ax-b{\|}_{2}+𝓡(x)$$

where 𝓡 is a regularization function (e.g., total variation). In practice, vendor-provided ordered-subset expectation-maximization (OSEM) or ToF-OSEM with point-spread-function (PSF) modeling are used for clinical imaging [36], [37]. In our experiments, we utilize clinical image reconstruction parameters for the GE Signa PET/MRI (Table II).

### B. Synthetic Sinogram Generation and Lesion Insertion

For a given system matrix A, a reconstructed PET image x can be projected into the sinogram domain by applying the forward model (7) to yield $y_{\text{simulated}}$. The forward projection tool provided with the Duet to toolbox (v02.03, GE Healthcare) performs this operation on a synthetic volume of dimension equal to the reconstructed volume, to generate a synthetic lesion sinogram that is added to the sinogram corresponding to x. Image reconstruction can then be performed on this “lesion-inserted” sinogram, as if it were the real sinogram, using a variety of methods (e.g., ToF, PSF, and regularization).

**Fig. 4.**
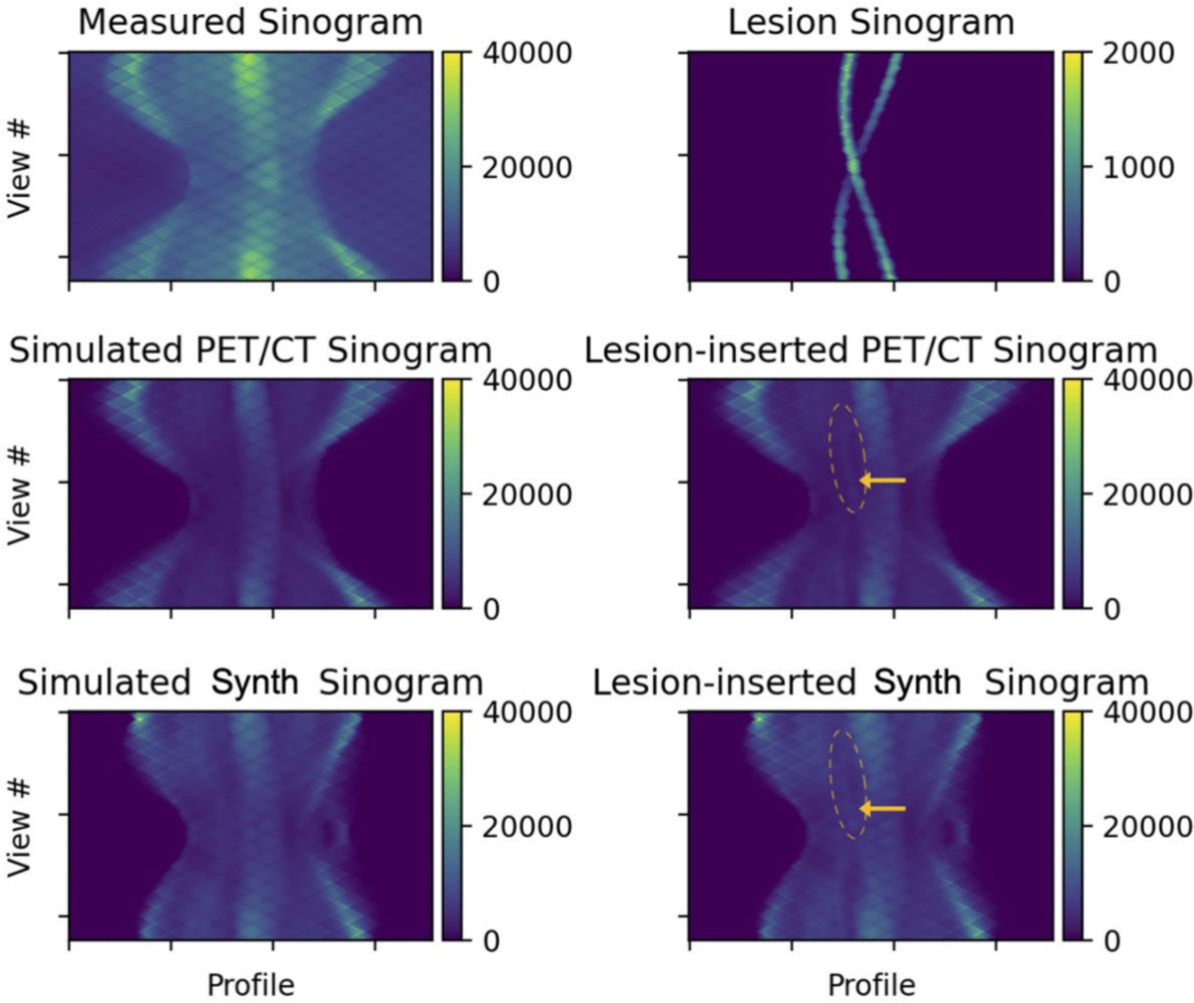
Measured and simulated sinograms representing different PET sources with corresponding synthetically inserted lesion sinograms. The annotation (yellow arrow) highlights a region affected by lesion insertion.

We exploit this mechanism to generate sPET sinogram data from domain-translated sPET imagery. However, as Duetto currently does not incorporate scatter simulation, we perform reconstructions with scatter estimation and correction turned off. As this introduces an additional discrepancy between real PET and sPET reconstructions, in both cases we start by forward projecting a 3-D “source” volume $x_{\text{source}}$ to yield a simulated ToF-sinogram that is subsequently inserted with synthetic lesions (Fig. 4).

### C. Quantification Experiment Summary

The pelvic CTAC versus MRAC FDG-PET reconstruction and SUV quantification experiment can be summarized as follows.

1. Forward project $x_{\text{source}}$ using a registered CT-based attenuation map to yield sinogram $y_{\text{simulated}}$. To evaluate the applicability of different sPET sources for this pipeline, we choose $x_{\text{source}}$ as follows.
  - *Real PET* $x_{real}$: The true patient activity distribution, corresponding to measured patient sinogram $y_{\text{real}}$.
  - *Reconstructed Patient Phantom* $x_{live}$: A PET/CT image volume, reconstructed from measured PET sinogram data with a CT-based attenuation map.
  - *Uniform SUV∼1* $x_{uniform}$: We threshold a T1-weighted postcontrast MRI volume to define a body-mask filled with activity corresponding to SUV 1.
  - *Synthetic sPET* $x_{synthetic}$: An sPET volume generated from a T1-weighted postcontrast MRI using the aforementioned 3-D UNet.
2. Forward project synthetic lesions specified by a 3-D volume $x_{\text{lesion}}$ to yield $y_{\text{lesion-simulated}}$. In our experiments, a board-certified radiologist annotated four sites for lesion insertion in each pelvic MR exam: a) in the acetabulum; b) sacrum; c) rectum; and d) lymph nodes. These locations were specifically identified to challenge the ability of MR-based reconstruction to reproduce activity surrounded by soft tissue and bone. For each location, a spherical lesion with diameter 12 mm and activity corresponding to SUV 8 was added to a zero-filled $x_{\text{lesion}}$ volume.
3. Reconstruct lesion-inserted sinograms using vendor-provided CTAC and MRAC methods (with parameters specified in Section III-A), resulting in PET images ${\hat{x}}_{\text{CT}}$ and ${\hat{x}}_{\text{MR}}$, respectively, for each $x_{\text{source}}$.
4. Evaluate voxel-wise and regional absolute and relative error between ${\hat{x}}_{\text{CT}}$ and ${\hat{x}}_{\text{MR}}$ in each lesion volume of interest (VOI) for each $x_{\text{source}}$ for each exam. Evaluation within each VOI can also provide a quantitative measure of accuracy, since the activity was synthetically inserted.

In particular, we evaluate the ability of different synthetic sinograms (corresponding to a choice of $x_{\text{source}}$) to reproduce the CTAC versus MRAC “quantification error” ${\text{Δ}}_{\text{quant}}$, normally estimated using real measured PET sinogram data. We quantify this by computing and comparing *deviation of error* in mean-, max-, and peak-SUV between ${\hat{x}}_{\text{CT}}$ and ${\hat{x}}_{\text{MR}}$ for each $x_{\text{source}}$ in each VOI compared to using real PET sinogram data. That is, for each $x_{\text{source}}$ we compute

(9) $${\text{Δ}}_{\text{quant,source}}=\|\text{quant}\left({\hat{x}}_{\text{CT}}[\text{V}]\right)-\mathrm{quant}\left({\hat{x}}_{\text{MR}}[\text{V}]\right)){\|}_{1}$$

(10) $$\delta_{\text{quant, source}}=\left|{\text{Δ}}_{\text{quant, true}}-{\text{Δ}}_{\text{quant, source}}\right|$$

(11) $$\gamma_{\text{quant, source}}=\frac{\left|{\text{Δ}}_{\text{quant, true}}-{\text{Δ}}_{\text{quant, source}}\right|}{{\text{Δ}}_{\text{quant, true}}}\times 100\%$$

where V represents an indicator function for voxels in a VOI, quant represents the mean-, max-, or peak-SUV computation in a VOI, we take ${\text{Δ}}_{\text{true}}$ as the corresponding mean-, max-, or peak-SUV quantification error computed using the reconstruction patient phantom $x_{\text{live}}$ as the source, and δ and γ represent absolute and relative quantification error, respectively. To benchmark systematic error arising from the reconstruction and reprojection in the experimental procedure, we also compare to the quantification error arising from using measured sinogram data $y_{\text{real}}$ corresponding to the true patient distribution $x_{\text{real}}$ (i.e., following the standard approach in [16]).

**Fig. 5.**
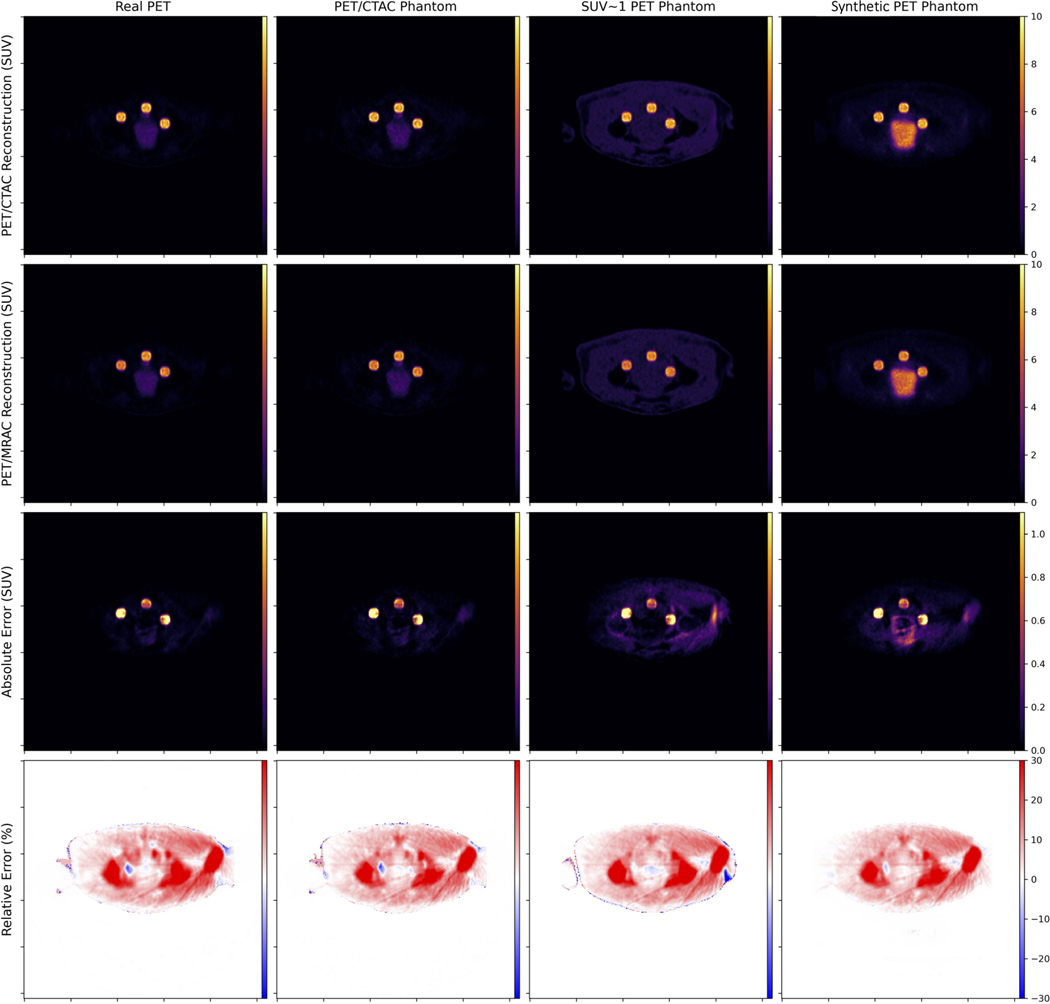
Example evaluation of synthetically inserted lesions into 3-D reconstructions using various PET data sources (anterior is superior in our presentations). For the PET data source (columns), we compute a reconstruction using CTAC and MRAC, and compute the absolute and relative errors for each slice. Shown here is a single slice from a single patient with contributions from three synthetically inserted lesions. The error in the sPET prediction is considerably lower than using the phantom with SUV∼1, and has a similar distribution to using real PET data.

For each patient exam, we select five different VOIs: lesion voxels corresponding to the four annotated regions (acetabulum, sacrum, rectum, and lymph), and “background,” representing all nonzero voxels outside of the synthetic lesions. Quantification error is computed for each VOI by comparing mean-, max-, and peak-SUV between CTAC-based and MRAC-based reconstructions. Subsequently, we compare the quantification error predicted by each PET data source to that predicted by the aforementioned reconstructed PET/CTAC live phantom. The absolute error is quantified for the background pixels, but the relative error is not since many voxels are devoid of any activity, positively skewing (overestimating) the mean relative error computation. In lieu of individual regions within the pelvis, the relative error in background voxels is better evaluated qualitatively by comparing slices in the transverse plane (Fig. 5).

### D. Results on Pelvic ^18^F-FDG PET/MR/CT Datasets

Numerical results presented in Tables III and IV indicate that domain-translated MR-based sPET can achieve low absolute and relative deviation in quantification error compared to the quantification error predicted by the live PET/CTAC phantom source for synthetically inserted pelvic lesions. Table III shows that sPET-based evaluation to compare CTAC and MRAC-based reconstruction achieves SUV errors that were very similar to the measured PET-based evaluation across inserted lesions and in the background. The percent quantification errors in Table IV shows that sPET-based evaluation was more similar to measured PET-based evaluation than uniform SUV∼1-based evaluation, outperforming for mean-SUV evaluations across lesion types. This suggests the applicability of synthetic sPET as a suitable replacement for real measured PET in PET-SUV quantification tasks. In the supplementary material (Figs. 6 and 7), we provide the Bland–Altman plots that compare the CTAC-versus MRAC error computed by the various types of phantoms and the Live Phantom. Each column represents a different sPET phantom. Each row represents a different error metric (absolute error or relative error in mean-SUV, peak-SUV, or max-SUV). This analysis shows no significant differences between sPET and measured PET using the aforementioned figures of merit.

## IV. Discussion

Overall, we have shown that MR-derived synthetic FDG-PET accurately captures the background physiologic distribution of PET imagery, creating images with realistic spatial distributions, and that it can be combined with synthetic lesion insertion to provide data for the evaluation of PET quantification methods. However, the main limitation we observed is that it is smoother than corresponding full-dose imagery, perhaps due to the implicit regularization properties of convolutional networks (e.g., exploited by DIP techniques [22]). While this is desirable for enhancing low-dose or noisy PET/MRI, it is not entirely beneficial for our application due to mismatches in the intensity distribution used in the quantification experiment.

This is a good opportunity for future works that make use of GANs, which may seek to better match the statistical distribution of sPET and PET to increase its realism, rather than simply regressing by value. Note that a pure GAN approach based on noise vectors is not valid here because it may not provide anatomic conformity between the MRI and sPET image, which is important to maintain for PET/MRI reconstruction algorithm research. Instead, adversarial losses may be added to the existing approach to increase realism and to help reduce artifacts in the regions where variable uptake is expected, or where patch-based inference lacks sufficient context to prevent gridding or stitching artifacts (although the effect of these artifacts is often reduced after forward projection to sinogram space). In this respect, the physics-based tomographic LOR loss utilized in this article not only works to increase the realism of sPET but also improves its quantitative accuracy.

We believe that such physics-based approaches are crucial for the development of quantitative imaging and dataset generation techniques based on neural networks. While the tomographic LOR loss used in this work improves the quantitative error rates and qualitative realism (partially captured by SSIM) associated with sPET, advanced physics-based modeling could further improve both the realism as well as the applicability of the developed approach to more PET/MRI systems, e.g., by utilizing their system matrix to optimize directly in the singoram domain, or measure congruence after image reconstruction.

Results from the downstream PET SUV quantification experiment indicate that sPET can serve as an adequate surrogate for real data in an MRAC versus CTAC quantification experiments. This experiment also indicates that the PET background distribution does not significantly impact quantification performance when using synthetically inserted lesions and without any scatter and randoms simulation. Thus, further investigation of a more complete reconstruction is required to determine whether the PET background distribution affects quantification for real lesions. Based on the realistic appearance of sPET, we believe it will be an important tool in evaluations when accurate background distribution is required.

Although in some cases the estimate based on sPET underestimates the benchmark (reconstructed patient phantom) error, the strong agreement over a number of exams (N=20) and lesions indicates that sPET may be used as a qualification method when a large number of exams is required. This is precisely the domain for which sPET was designed, as large MRI/CT databases can be retrospectively utilized to establish a large sPET/MRAC/CTAC dataset for scanner and algorithm qualification. Interestingly, in many cases the quantification error predicted by the synthetic sPET phantom more closely matches the quantification error predicted using real PET sinogram data, compared to even that of the live PET/CT phantom. However, for most VOIs and phantom types, the deviation in quantification error is minimal. Here, synthetic sPET has an advantage over the uniform SUV∼1 phantom, because it a represents a realistic, anthropomorphic PET uptake pattern.

One limitation of our methodology is that it does not directly model noise or count statistics associated with PET data collection, which has been shown to impact the performance of PET reconstruction algorithms [38], [39], [40]. To address this, we note that the MR-based sPET images proposed in this article can be treated simply as an ideal source volume and, thus, readily combined with Monte Carlo PET simulators, such as with GATE [25], SIMSPET [41], or SimPET [3]. An alternative data-driven approach to address this issue may be to utilize a adversarial training, which can increase the realism of sPET, thereby indirectly capturing statistical noise properties of PET acquisition in the image domain.

## V. Future Work

Detailing the generation of sPET from 3-D MRI and, importantly, demonstrating its utility in downstream qualification research, opens the path to new research directions that can enable us to study new PET image reconstruction algorithms that can address important clinical questions. For example, virtual PET clinics have been previously proposed as a technique to evaluate PET detector systems and patient studies in a virtual simulation environment [2]. This could also be extended to address 4D PET/CT and PET/MRI modalities [42], enabling new approaches to diagnose cancers, such as the identification of recurrent gliomas using FET PET [43], [44]. In another vein, sPET can also be used to directly improve image reconstruction algorithms themselves, e.g., by generation of a deep learning prior image that can help regularize PET image reconstruction [45]. These applications provide a strong motivation for future work in curating large databases of PET/MRI with multiple MRI contrasts and PET radiotracer images, which could mirror and complement the impact of other synthetic MRI [46], [47]. In this respect, the methods developed in this article provide the framework and context necessary for such development.

## VI. Conclusion

In conclusion, we have demonstrated a method using deep learning to generate realistic, synthetic whole-body PET data from MRI, and that it is a suitable substitute for real PET data in a reconstruction evaluation task. The sPET data, which mimics physiologic tracer distribution, can be combined with synthetic lesion insertion to mimic abnormal regions of high update. We demonstrated its equivalent performance to real PET data for comparing CTAC and MRAC for PET reconstruction, and believe this result combined with the apparent realism of the synthetic images will make this method broadly applicable for evaluating the robustness of PET/MRI reconstructions and component techniques, including attenuation correction, scatter correction, and MR-guided reconstruction algorithms, using large and diverse patient datasets.

All source code for this article, including sPET training code, PET reconstruction wrappers, and quantification experiments, is available freely at: https://gitlab.com/abhe/SyntheticPET-TRPMS22https://gitlab.com/abhe/SyntheticPET-TRPMS22

## Supplementary Material

supplemental

## Figures and Tables

**TABLE I:** Test-Set Performance ± 1 Stdev Using Different Supervised MR-to-PET Domain Translation Training Objectives

Objective	*MAE* (Eq. 4)	*MRAE* (Eq. 5)	*3D-SSIM* (Eq. 6)
MAE (Eq. 4)	0.090 ± 0.021	0.654 ± .074	0.473 ± 0.073
J (Eq. 2)	0.083 ± 0.031	0.487 ±.145	0.863 ± 0.089
$J_{\text{total}}$ (Eq. 1)	**0.066** ± 0.026	**0.369** ± .092	**0.938** ± 0.060

**TABLE II:** Summary of PET Reconstruction Hyperparameters

Parameter	Value
Objective Function	ToF-OSEM-PSF
Subsets	28
Iterations	2
Transverse Filter (FWHM)	2.0 mm
Axial Filter (FWHM)	4.0 mm
Transverse Field-of-View (FOV)	600 mm
Transverse matrix size	[256,256]
# Projection Angles / Views	180

**TABLE III:** Absolute Errors Between CTAC- and MRAC-Based Reconstructions for Various Real and sPET Data Sources

	*Average Difference in Mean-SUV between PET/CTAC and PET/MRAC Reconstructions in*					
PET Data Source	*All Lesions*	*Lesion (Acetabulum)*	*Lesion (Sacrum)*	*Lesion (Rectum)*	*Lesion (Lymph)*	*Background*
$x_{\text{real}}$	0.474	0.630	0.627	0.271	0.410	0.011

$x_{\text{live}}$	0.485	0.644	0.647	0.276	0.422	0.012
$x_{\text{uniform}}$	0.505	0.694	0.688	0.262	0.429	0.033
$x_{\text{synthetic}}$	0.498	0.658	0.666	0.277	0.439	0.024
	*Average Difference in Max-SUV between PET/CTAC and PET/MRAC Reconstructions in*					
PET Data Source	*All Lesions*	*Lesion (Acetabulum)*	*Lesion (Sacrum)*	*Lesion (Rectum)*	*Lesion (Lymph)*	*Background*

$x_{\text{real}}$	0.524	0.985	1.015	0.353	0.606	0.539

$x_{\text{live}}$	0.484	0.995	1.043	0.348	0.608	0.545
$x_{\text{uniform}}$	0.586	1.017	1.053	0.305	0.602	0.586
$x_{\text{synthetic}}$	0.512	0.991	1.022	0.328	0.588	0.476
	*Average Difference in Peal-SUV between PET/CTAC and PET/MRAC Reconstructions in*					
PET Data Source	*All Lesions*	*Lesion (Acetabulum)*	*Lesion (Sacrum)*	*Lesion (Rectum)*	*Lesion (Lymph)*	*Background*

$x_{\text{real}}$	0.461	0.912	0.945	0.310	0.600	0.488

$x_{\text{live}}$	0.371	0.929	0.970	0.302	0.592	0.457
$x_{\text{uniform}}$	0.540	0.979	1.006	0.302	0.607	0.540
$x_{\text{synthetic}}$	0.484	0.943	0.976	0.335	0.555	0.406

**TABLE IV:** Deviation in Predicted Quantification Error (Lower Is Better) for Various Real and sPET Data Sources. ± Indicates One Standard Deviation

	*Percent Error in Mean-SUV compared to Baseline (* $x_{live}$ *) in*			
PET Data Source	*Lesion (Acetabulum)*	*Lesion (Sacrum)*	*Lesion (Rectum)*	*Lesion (Lymph)*
$x_{\text{real}}$	2.48 ± 2.10	7.56 ± 8.50	3.85 ± 4.22	4.06 ± 3.16

$x_{\text{uniform}}$	7.75 ± 2.20	7.34 ± 4.57	22.09 ± 2.86	**2.47** ± 1.46
$x_{\text{synthetic}}$	**2.94** ± 1.50	**5.54** ± 3.80	**15.88** ± 2.07	6.17 ± 1.59
	*Percent Error in Max-SUV compared to Baseline (* $x_{live}$ *) in*			
PET Data Source	*Lesion (Acetabulum)*	*Lesion (Sacrum)*	*Lesion (Rectum)*	*Lesion (Lymph)*

$x_{\text{real}}$	7.57 ± 6.48	8.23 ± 4.94	80.61 ± 14.41	16.16 ± 1.58

$x_{\text{uniform}}$	8.56 ± 6.46	**9.84** ± 7.05	**66.07** ± 14.54	17.74 ± 1.31
$x_{\text{synthetic}}$	**3.42** ± 3.49	13.53 ± 2.14	121.53 ± 15.78	**12.88** ± 1.78
	*Percent Error in Peak-SUV compared to Baseline (* $x_{live}$ *) in*			
PET Data Source	*Lesion (Acetabulum)*	*Lesion (Sacrum)*	*Lesion (Rectum)*	*Lesion (Lymph)*

$x_{\text{real}}$	2.56 ± 2.07	15.96 ± 18.71	29.01 ± 58.60	5.28 ± 3.82

$x_{\text{uniform}}$	2.73 ± 0.81	19.56 ± 29.70	**34.53** ± 40.99	**2.17** ± 2.42
$x_{\text{synthetic}}$	**1.98** ± 1.48	**9.60** ± 11.61	92.30 ± 19.34	3.20 ± 1.93

## References

[1] Hope TA , “Summary of the first ISMRM–SNMMI workshop on PET/MRI: Applications and limitations,” J. Nucl. Med, vol. 60, no. 10, pp. 1340–1346, 2019. 31123099 10.2967/jnumed.119.227231 PMC6785790

[2] Abadi E. , “Virtual clinical trials in medical imaging: A review,” J. Med. Imag, vol. 7, no. 4, 2020, Art. no. 42805. 10.1117/1.JMI.7.4.042805 PMC7148435 32313817

[3] Paredes-Pacheco J. , “SimPET—An open online platform for the Monte Carlo simulation of realistic brain PET data. Validation for 18F-FDG scans,” Med. Phys, vol. 48, no. 5, pp. 2482–2493, 2021. 33713354 10.1002/mp.14838 PMC8252452

[4] MacFarlane CR, “ACR accreditation of nuclear medicine and PET imaging departments,” J. Nucl. Med Technol., vol. 34, no. 1, pp. 18–24, 2006. 16517965

[5] Ziegler S, Jakoby BW, Braun H, Paulus DH, and Quick HH, “NEMA image quality phantom measurements and attenuation correction in integrated PET/MR hybrid imaging,” EJNMMI Phys., vol. 2, no. 1, pp. 1–14, 2015. 26501819 10.1186/s40658-015-0122-3 PMC4542864

[6] Wollenweber SD , “Evaluation of an atlas-based PET head attenuation correction using PET/CT & MR patient data,” IEEE Trans. Nucl. Sci, vol. 60, no. 5, pp. 3383–3390, Oct. 2013.

[7] Wollenweber SD , “Comparison of 4-class and continuous fat/water methods for whole-body, MR-based PET attenuation correction,” IEEE Trans. Nucl. Sci, vol. 60, no. 5, pp. 3391–3398, Oct. 2013.

[8] Torrado-Carvajal A. , “Fast patch-based pseudo-CT synthesis from T1-weighted MR images for PET/MR attenuation correction in brain studies,” J. Nucl. Med, vol. 57, no. 1, pp. 136–143, 2016. 26493204 10.2967/jnumed.115.156299

[9] Defrise M, Rezaei A, and Nuyts J, “Simultaneous reconstruction of attenuation and activity in TOF-PET: Analysis of the convergence of the MLACF algorithm,” in Proc. Fully 3D Meeting, 2013, pp. 1–4.

[10] Rezaei A, Deroose CM, Vahle T, Boada F, and Nuyts J, “Joint reconstruction of activity and attenuation in time-of-flight PET: A quantitative analysis,” J. Nucl. Med, vol. 59, no. 10, pp. 1630–1635, 2018. 29496982 10.2967/jnumed.117.204156 PMC6167531

[11] Leynes AP , “Zero-echo-time and Dixon deep pseudo-CT (ZeDD CT): Direct generation of pseudo-CT images for pelvic PET/MRI attenuation correction using deep convolutional neural networks with multiparametric MRI,” J. Nucl. Med, vol. 59, no. 5, pp. 852–858, 2018. 29084824 10.2967/jnumed.117.198051 PMC5932530

[12] Gong K, Yang J, Kim K, El Fakhri G, Seo Y, and Li Q, “Attenuation correction for brain PET imaging using deep neural network based on Dixon and ZTE MR images,” Phys. Med. Biol, vol. 63, no. 12, 2018, Art. no. 125011. 10.1088/1361-6560/aac763 PMC6031313 29790857

[13] Leynes AP , “Bayesian deep learning uncertainty estimation and pseudo-CT prior for robust maximum likelihood estimation of activity and attenuation (UpCT-MLAA) in the presence of metal implants for simultaneous PET/MRI in the pelvis,” 2020, arXiv:2001.03414.

[14] Chen Y. , “Deep learning-based T1-enhanced selection of linear attenuation coefficients (DL-TESLA) for PET/MR attenuation correction in dementia neuroimaging,” Magn. Reson. Med, vol. 86, no. 1, pp. 499–513, 2021. 33559218 10.1002/mrm.28689 PMC8091494

[15] Chandramohan D. , “Bone material analogues for PET/MRI phantoms,” Med. Phys, vol. 47, no. 5, pp. 2161–2170, 2020. 32034945 10.1002/mp.14079 PMC7901472

[16] Catana C. , “A path to qualification of PET/MR scanners for multicenter brain imaging studies: Evaluation of MR-based attenuation correction methods using a patient phantom,” J. Nucl. Med, vol. 63, no. 4, pp. 615–621, 2021. 34301784 10.2967/jnumed.120.261881 PMC8973286

[17] Laforest R. , “Harmonization of PET image reconstruction HyperParameters in simultaneous PET/MRI,” Eur. J. Nucl. Med. Mol. Imag. Phys, vol. 8:75, pp. 1–22, May 2020, doi: 10.21203/rs.3.rs-29815/v2. [Online]. Available: https://ejnmmiphys.springeropen.com/counter/pdf/10.1186/s40658-021-00416-0.pdf

[18] Armanious K, Jiang C, Abdulatif S, Küstner T, Gatidis S, and Yang B, “Unsupervised medical image translation using cycle-MedGAN,” in Proc. 27th Eur. Signal Process. Conf. (EUSIPCO), 2019, pp. 1–5

[19] Yang Q. , “MRI cross-modality image-to-image translation,” Sci. Rep, vol. 10, no. 1, pp. 1–18, 2020. 32111966 10.1038/s41598-020-60520-6 PMC7048849

[20] Ouyang J, Chen KT, Gong E, Pauly J, and Zaharchuk G, “Ultra-low-dose PET reconstruction using generative adversarial network with feature matching and task-specific perceptual loss,” Med. Phys, vol. 46, no. 8, pp. 3555–3564, 2019. 31131901 10.1002/mp.13626 PMC6692211

[21] Xu J, Gong E, Ouyang J, Pauly J, Zaharchuk G, and Han S, “Ultra-low-dose 18F-FDG brain PET/MR denoising using deep learning and multi-contrast information,” in Proc. Med. Image Process, 2020, Art. no. 113131P.

[22] Gong K, Catana C, Qi J, and Li Q, “PET image reconstruction using deep image prior,” IEEE Trans. Med. Imag, vol. 38, no. 7, pp. 1655–1665, Jul. 2019. 10.1109/TMI.2018.2888491 PMC6584077 30575530

[23] Yokota T, Kawai K, Sakata M, Kimura Y, and Hontani H, “Dynamic pet image reconstruction using nonnegative matrix factorization incorporated with deep image prior,” in Proc. IEEE/CVF Int. Conf. Comput. Vis, 2019, pp. 3126–3135.

[24] Zaharchuk G. and Davidzon G, “AI for optimization and interpretation of PET/CT and PET/MR images,” Seminars Nucl. Med, vol. 51, no. 2, pp. 134–142, 2020. 10.1053/j.semnuclmed.2020.10.001 33509370

[25] Jan S. , “GATE: A simulation toolkit for PET and SPECT,” Phys. Med. Biology, vol. 49, no. 19, pp. 4543–4561, 2004. 10.1088/0031-9155/49/19/007 PMC3267383 15552416

[26] España S, Herraiz J, Vicente E, Vaquero JJ, Desco M, and Udías JM, “PeneloPET, a Monte Carlo PET simulation tool based on PENELOPE: Features and validation,” Phys. Med. Biol, vol. 54, no. 6, p. 1723, 2009. 19242053 10.1088/0031-9155/54/6/021

[27] Zhang C, Cheng J, Liu J, Pang J, Huang Q, and Tian Q, “Beyond explicit codebook generation: Visual representation using implicitly transferred codebooks,” IEEE Trans. Image Process, vol. 24, pp. 5777–5788, 2015. 26441449 10.1109/TIP.2015.2485783

[28] Gorgolewski K. , “Nipype: A flexible, lightweight and extensible neuroimaging data processing framework in Python,” Front. Neuroinform, vol. 5, p. 13, Aug. 2011. 21897815 10.3389/fninf.2011.00013 PMC3159964

[29] Zuiderveld K, “Contrast limited adaptive histogram equalization,” in Graphics Gems IV. Boston, MA, USA: AP Professional, 1994, pp. 474–485.

[30] Byrd D, Wollenweber S, Alessio A, Stearns C, and Kinahan PE, “Metrics for assessing quantitative accuracy of PET/CT systems,” Eur. J. Nucl. Med. Mol. Imag, vol. 43, pp. S518–S519, Oct. 2016.

[31] Çiçek Ö, Abdulkadir A, Lienkamp SS, Brox T, and Ronneberger O, “3D U-Net: Learning dense volumetric segmentation from sparse annotation,” in Proc. Int. Conf. Med. Image Comput. Comput.-Assist. Intervention, 2016, pp. 424–432.

[32] Alom MZ, Hasan M, Yakopcic C, Taha TM, and Asari VK, “Recurrent residual convolutional neural network based on U-Net (R2U-Net) for medical image segmentation,” 2018, arXiv:1802.06955.

[33] Wang Z, Simoncelli EP, and Bovik AC, “Multiscale structural similarity for image quality assessment,” in Proc. 37th Asilomar Conf. Signals, Syst. Comput., vol. 2, 2003, pp. 1398–1402.

[34] Bach-Gansmo T, Dybvik J, Adamsen T, and Naum A, “Variation in urinary excretion of FDG, yet another uncertainty in quantitative PET,” Acta Radiologica Short Rep., vol. 1, no. 8, pp. 1–3, 2012. 10.1258/arsr.2012.120038 PMC3738358 23986849

[35] Hamdi M. , “Evaluation of attenuation correction in PET/MRI with synthetic lesion insertion,” J. Med. Imag, vol. 8, no. 5, 2021, Art. no. 56001. 10.1117/1.JMI.8.5.056001 PMC8451234 34568511

[36] Wadhwa P. , “PET image reconstruction using physical and mathematical modelling for time of flight PET-MR scanners in the STIR library,” Methods, vol. 185, pp. 110–119, Jan. 2021. 32006678 10.1016/j.ymeth.2020.01.005

[37] Rogasch JM , “Reconstructed spatial resolution and contrast recovery with Bayesian penalized likelihood reconstruction (Q. Clear) for FDG-PET compared to time-of-flight (TOF) with point spread function (PSF),” EJNMMI Phys., vol. 7, no. 1, pp. 1–14, 2020. 31925574 10.1186/s40658-020-0270-y PMC6954158

[38] Tong S, Alessio A, and Kinahan P, “Noise and signal properties in PSF-based fully 3D PET image reconstruction: An experimental evaluation,” Phys. Med. Biol, vol. 55, no. 5, p. 1453, 2010. 20150683 10.1088/0031-9155/55/5/013 PMC2890317

[39] Lodge MA, Chaudhry MA, and Wahl RL, “Noise considerations for PET quantification using maximum and peak standardized uptake value,” J. Nucl. Med, vol. 53, no. 7, pp. 1041–1047, 2012. 22627001 10.2967/jnumed.111.101733 PMC3417317

[40] Rahmim A. and Tang J, “Noise propagation in resolution modeled PET imaging and its impact on detectability,” Phys. Med. Biol, vol. 58, no. 19, p. 6945, 2013. 24029682 10.1088/0031-9155/58/19/6945 PMC3866837

[41] Guérin B. and El Fakhri G, “Realistic PET Monte Carlo simulation with pixelated block detectors, light sharing, random coincidences and deadtime modeling,” IEEE Trans. Nucl. Sci, vol. 55, no. 3, pp. 942–952, Jun. 2008. 19079776 10.1109/TNS.2008.924064 PMC2600659

[42] Tsoumpas C. and Gaitanis A, “Modeling and simulation of 4D PET-CT and PET-MR images,” PET Clin., vol. 8, no. 1, pp. 95–110, 2013. 27157818 10.1016/j.cpet.2012.10.003

[43] Debus C. , “Impact of 18F-FET PET on target volume definition and tumor progression of recurrent high grade glioma treated with carbon-ion radiotherapy,” Sci. Rep, vol. 8, no. 1, pp. 1–13, 2018. 29740097 10.1038/s41598-018-25350-7 PMC5940831

[44] Lohmeier J, Bohner G, Siebert E, Brenner W, Hamm B, and Makowski MR, “Quantitative biparametric analysis of hybrid 18F-FET PET/MR-neuroimaging for differentiation between treatment response and recurrent glioma,” Sci. Rep, vol. 9, no. 1, pp. 1–9, 2019. 31601829 10.1038/s41598-019-50182-4 PMC6787240

[45] Rajagopal A, Dwork N, Hope TA, and Larson PE, “Enhanced PET/MRI reconstruction via dichromatic interpolation of domain-translated zero-dose PET,” in Proc. Phys. Med. Imag, 2021, Art. no. 115954B.

[46] Hagiwara A. , “Contrast-enhanced synthetic MRI for the detection of brain metastases,” Acta Radiologica Open, vol. 5, no. 2, 2016, Art. no. 2058460115626757. 10.1177/2058460115626757 PMC4765820 26962461

[47] Ji S, Yang D, Lee J, Choi SH, Kim H, and Kang KM, “Synthetic MRI: Technologies and applications in neuroradiology,” J. Magn. Reson. Imag, vol. 55, no. 4, pp. 1013–1025, 2020. 10.1002/jmri.27440 33188560

