# Supplementary material for: Synthetic PET via Domain Translation of 3-D MRI
Source: IEEE Trans Radiat Plasma Med Sci. Author manuscript; Available in PMC 2023 Jun 30. (PMC10311993; doi:10.1109/trpms.2022.3223275)
Supplement: supplemental [file NIHMS1888268-supplement-supplemental.pdf]

Supplementary Material

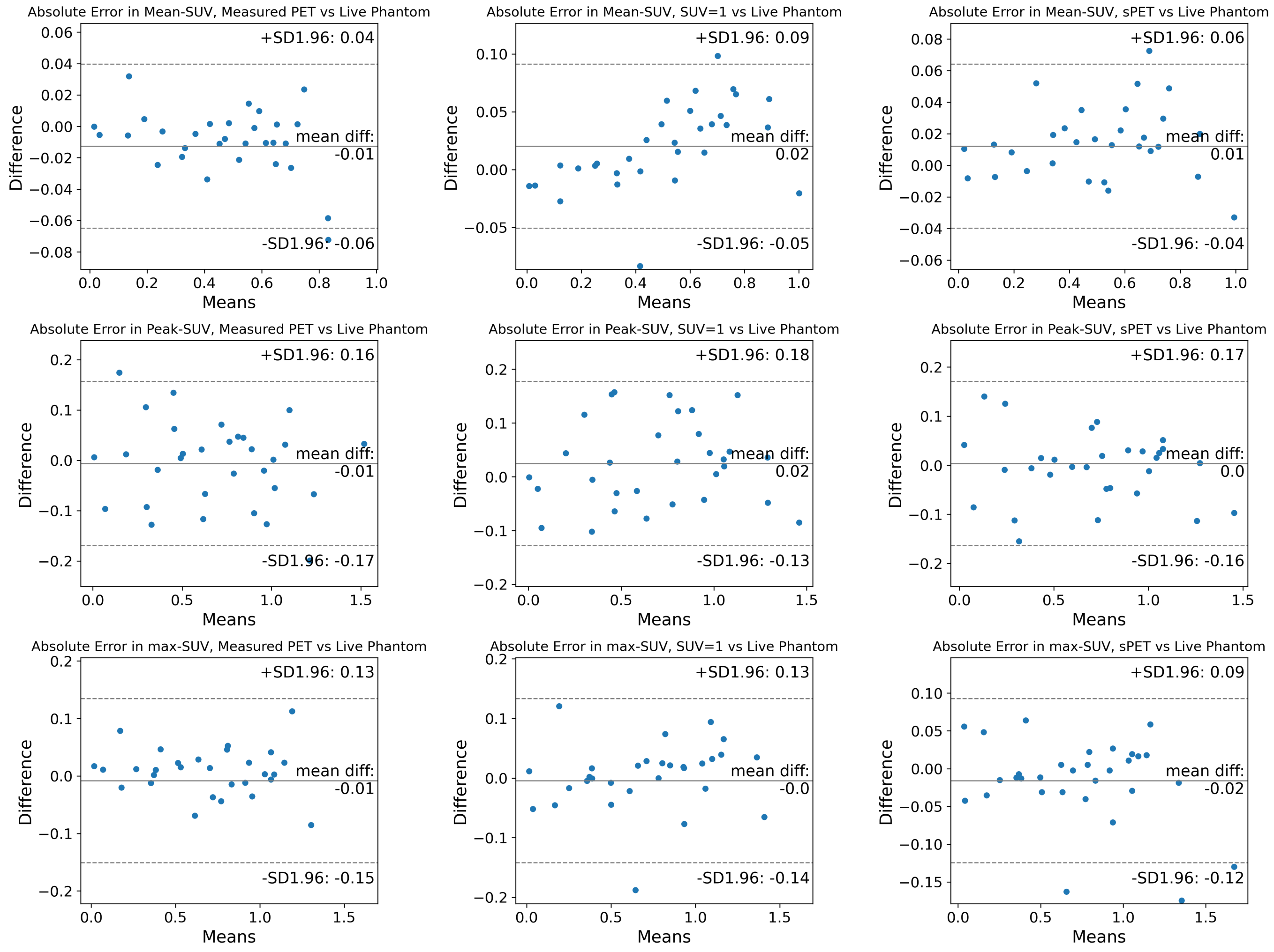

Fig. 6. Bland-Altman plots that compare the CTAC-vs-MRAC *Absolute Error* computed by the various types of phantoms and the Live Phantom. Each column represents a different synthetic PET phantom. Each row represents the error with respect to each metric of interest (mean-SUV, peak-SUV, or max-SUV).

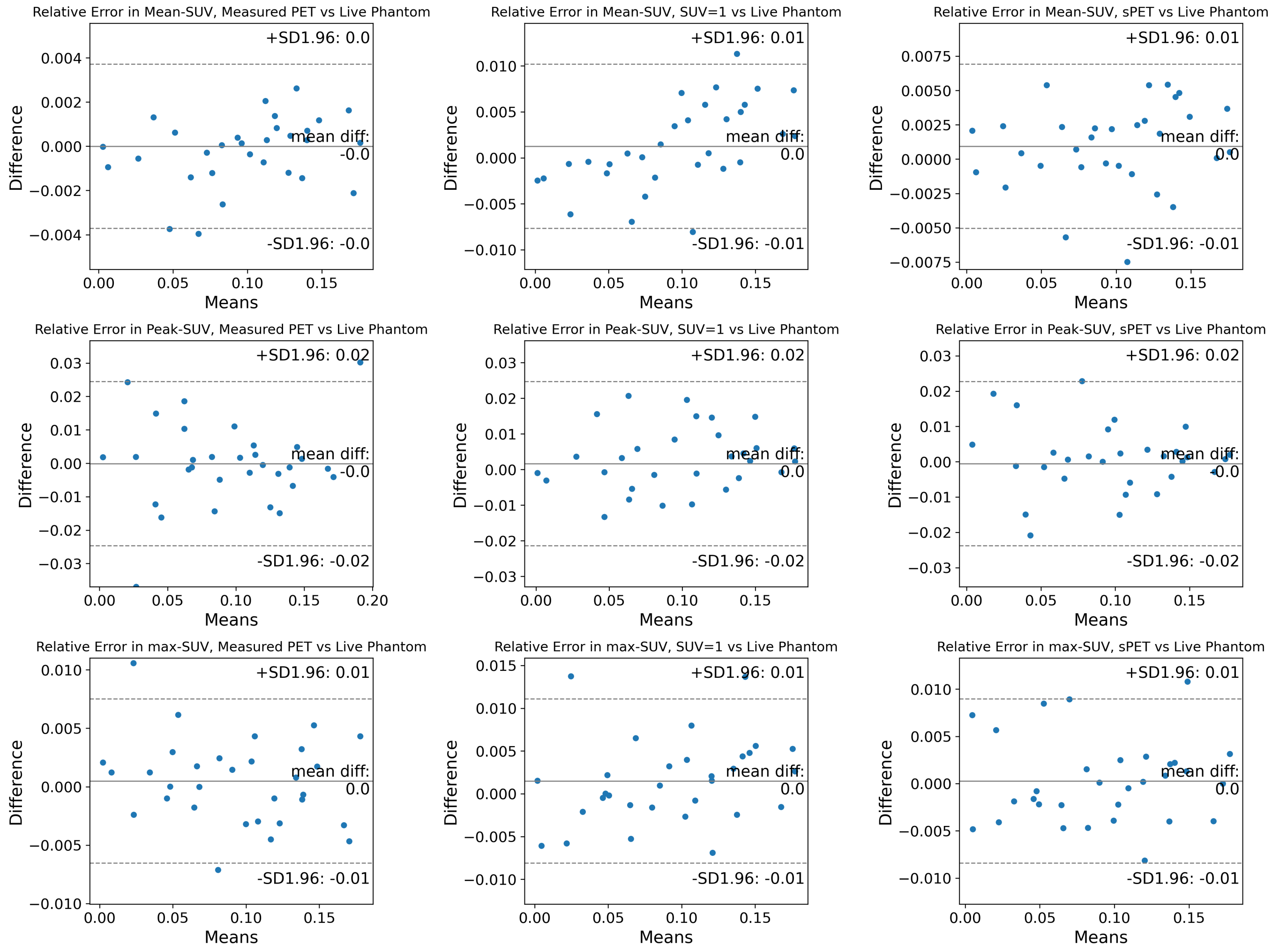

Fig. 7. Bland-Altman plots that compare the CTAC-vs-MRAC *Absolute Error* computed by the various types of phantoms and the Live Phantom. Each column represents a different synthetic PET phantom. Each row represents the error with respect to each metric of interest (mean-SUV, peak-SUV, or max-SUV).
